# Frequency of bystander exposure to antibiotics for enteropathogenic bacteria among young children in low-resource settings

**DOI:** 10.1073/pnas.2208972119

**Published:** 2022-08-29

**Authors:** Elizabeth T. Rogawski McQuade, Stephanie A. Brennhofer, Sarah E. Elwood, Timothy L. McMurry, Joseph A. Lewnard, Estomih R. Mduma, Sanjaya Shrestha, Najeeha Iqbal, Pascal O. Bessong, Gagandeep Kang, Margaret Kosek, Aldo A. M. Lima, Tahmeed Ahmed, Jie Liu, Eric R. Houpt, James A. Platts-Mills

**Affiliations:** ^a^Department of Epidemiology, Rollins School of Public Health, Emory University, Atlanta, GA 30322;; ^b^Division of Infectious Diseases & International Health, University of Virginia, Charlottesville, VA 22908;; ^c^Department of Public Health Sciences, University of Virginia, Charlottesville, VA 22908;; ^d^Division of Epidemiology, School of Public Health, University of California, Berkeley, Berkeley, CA 94720;; ^e^Haydom Global Health Research Center, Haydom Lutheran Hospital, Haydom, Tanzania;; ^f^Walter Reed/AFRIMS Research Unit, Nepal, Kathmandu, 44600, Nepal;; ^g^Aga Khan University, Karachi, 74800, Pakistan;; ^h^University of Venda, Thohoyandou, 0950, South Africa;; ^i^Center for Global Health Equity, University of Virginia, Charlottesville, VA 22908;; ^j^Christian Medical College, Vellore, 632004, India;; ^k^Asociación Benéfica PRISMA, Iquitos, 15088, Peru;; ^l^Universidade Federal do Ceara, Fortaleza, 60020-181, Brazil;; ^m^International Centre for Diarrhoeal Disease Research, Bangladesh, Dhaka, 1212, Bangladesh;; ^n^School of Public Health, Qingdao University, Qingdao, Shandong, 266071, China

**Keywords:** antimicrobial resistance, antibiotics, bystander exposure, enteric infections, children

## Abstract

Children in low-resource settings carry enteric pathogens asymptomatically and are frequently treated with antibiotics, resulting in opportunities for pathogens to be exposed to antibiotics when not the target of treatment (i.e., bystander exposure). We quantified the frequency of bystander antibiotic exposures for enteric pathogens and estimated associations with resistance among children in eight low-resource settings. We analyzed 15,697 antibiotic courses from 1,715 children aged 0 to 2 y from the MAL-ED birth cohort. We calculated the incidence of bystander exposures and attributed exposures to respiratory and diarrheal illnesses. We associated bystander exposure with phenotypic susceptibility of E. coli isolates in the 30 d following exposure and at the level of the study site. There were 744.1 subclinical pathogen exposures to antibiotics per 100 child-years. Enteroaggregative *Escherichia coli* was the most frequently exposed pathogen, with 229.6 exposures per 100 child-years. Almost all antibiotic exposures for *Campylobacter* (98.8%), enterotoxigenic *E. coli* (95.6%), and typical enteropathogenic *E. coli* (99.4%), and the majority for *Shigella* (77.6%), occurred when the pathogens were not the target of treatment. Respiratory infections accounted for half (49.9%) and diarrheal illnesses accounted for one-fourth (24.6%) of subclinical enteric bacteria exposures to antibiotics. Bystander exposure of *E. coli* to class-specific antibiotics was associated with the prevalence of phenotypic resistance at the community level. Antimicrobial stewardship and illness-prevention interventions among children in low-resource settings would have a large ancillary benefit of reducing bystander selection that may contribute to antimicrobial resistance.

## Significance

Antimicrobial resistance is a pressing concern, and while antibiotic stewardship interventions are intended to limit unnecessary antibiotic exposures, including to asymptomatically carried pathogens (i.e., bystander exposure), the frequency and characteristics of these bystander exposures have not been well described. We quantified the frequency that bacterial enteric pathogens were exposed to antibiotics when not the target of treatment in a study of children in low-resource settings. Our analysis demonstrated that almost all enteropathogen exposures to antibiotics occurred when the bacteria were carried asymptomatically, and respiratory infections were responsible for the largest proportion of exposures. Interventions to reduce antibiotic use and the illnesses that prompt treatment could have the ancillary benefit of reducing selection pressure for antimicrobial resistance among pathogens carried asymptomatically.

Antibiotic use causes selection pressure for antimicrobial resistance (AMR), a growing global public health crisis that threatens to render antibiotics ineffective against many high-burden infections (1). Most of the concern is placed on the development of resistance in the target pathogen of treatment (i.e., the pathogen causing the treated illness). However, systemic treatment also results in antibiotic exposure for commensal bacteria and pathogens carried asymptomatically at the time of treatment (2). Selective pressure for resistance among organisms that are not the target pathogen has been called “bystander selection” (3, 4). While the public health relevance of resistance in nonpathogenic commensal organisms is less clear, bystander selection among pathogens carried asymptomatically at the time of treatment has direct consequences for the development of resistance in those pathogens (4). This type of selection has the potential to promote antibiotic-resistant disease in settings where subclinical carriage of pathogens is common.

Children in low-resource settings frequently carry enteric pathogens in the absence of diarrheal symptoms (5). Enteroaggregative *Escherichia coli* (EAEC), for example, was detected in nearly half (49%) of nondiarrheal stools collected in the first 2 y of life in the Etiology, Risk Factors and Interactions of Enteric Infections and Malnutrition and the Consequences for Child Health and Development Project (MAL-ED) birth cohort study conducted in South America, South Asia, and sub-Saharan Africa (6). *Campylobacter* and *Shigella*, which are on the World Health Organization priority pathogen list for concern for AMR (7), were detected in 28% (5) and 10% (8) of nondiarrheal stools, respectively. Antibiotic treatment is also highly common in these populations, with approximately five treatment courses per child-year observed in MAL-ED (9). Children were treated with more than one antibiotic course per child year for diarrhea alone (10), despite treatment guidelines that only recommend treatment for dysentery (11), which comprised less than 5% of diarrheal episodes (10). For these reasons, children in low-resource settings represent a unique population in which the burden of bystander selection on enteric pathogens could be particularly high.

Antimicrobial stewardship interventions to prevent antibiotic overuse and interventions to prevent illnesses that prompt antibiotic treatment, such as vaccines, could have the ancillary benefit of reducing bystander selection (12). However, the magnitude of this potential impact is unknown. A prior study quantified the proportion of antibiotic exposures for specific pathogens that were not related to the treatment of that pathogen based on modeled data from unrelated sources (3). The observational birth cohort study, MAL-ED, provides a unique opportunity to characterize bystander antibiotic exposure directly since testing for enteric pathogen carriage was conducted monthly in nondiarrheal stools from birth to 2 y of age, and antibiotic use was comprehensively documented during twice-weekly surveillance visits. Here, we aimed to quantify the absolute frequency of bystander antibiotic exposures for enteric bacterial pathogens carried asymptomatically at the time of treatment among children in MAL-ED. We compared the frequency of antibiotic exposures that occurred when the bacteria were the target pathogen to when they were bystanders and attributed bystander exposure to specific indications for treatment. We also identified child characteristics that were associated with bystander antibiotic exposures. Finally, we assessed the association between bystander antibiotic exposure and resistance both at the individual and the community level using *E. coli* as a model organism.

## Results

Among 1,715 children included in the analysis, caregivers reported 15,697 total antibiotic courses. The majority (*n* = 13,629, 86.8%) of antibiotic courses had a stool sample collected and tested by qPCR with valid results within the previous 30 d (Table 1). In sensitivity analyses, 66.3 and 25.4% of antibiotic courses had a stool sample collected and tested by qPCR with valid results within the previous 21 and 7 d, respectively. Overall, there were 22,161 distinct exposures of enteric pathogens to antibiotics that occurred when the pathogens were subclinical bystanders. Approximately half (*n* = 12,013; 54.2%) of exposures were to drug classes that are of particular concern for AMR (cephalosporins, fluoroquinolones, macrolides, and sulfonamides) (7). Of these classes, the highest frequency of exposure was to macrolides (20.1%). The number of antibiotic exposures varied by site, with relatively infrequent use in Brazil and South Africa. While antibiotic exposures were common in Tanzania, few were to drug classes of concern for AMR (21.2%).

**Table 1.** Bystander antibiotic exposures for asymptomatically carried enteric bacterial pathogens among 1,715 children enrolled in the MAL-ED cohort

	Dhaka, Bangladesh	Fortaleza, Brazil	Vellore, India	Bhaktapur, Nepal	Loreto, Peru	Naushero Feroze, Pakistan	Venda, South Africa	Haydom, Tanzania	Overall
Children included*	210	165	227	227	194	246	237	209	1,715
Total antibiotic courses	3,700	224	1,740	1,051	2,051	4,954	504	1,473	15,697
Total linked antibiotic courses†	3,233	148	1,537	966	1,905	4,268	395	1,177	13,629
Total bystander antibiotic exposures^‡^	6,131	125	2,700	1,399	3,445	5,270	332	2,759	22,161
Bystander antibiotic exposures by drug class^‡^									
Cephalosporins	1,347 (22.0)	31 (24.8)	849 (31.4)	219 (15.7)	106 (3.1)	1,780 (33.8)	2 (0.6)	11 (0.4)	4,345 (19.6)
Fluoroquinolones	621 (10.1)	0 (0.0)	159 (5.9)	80 (5.7)	156 (4.5)	118 (2.2)	3 (0.9)	6 (0.2)	1,143 (5.2)
Macrolides	2,532 (41.3)	5 (4.0)	226 (8.4)	276 (19.7)	1,001 (29.1)	262 (5.0)	21 (6.3)	122 (4.4)	4,445 (20.1)
Sulfonamides	28 (0.5)	6 (4.8)	262 (9.7)	137 (9.8)	616 (17.9)	556 (10.6)	27 (8.1)	448 (16.2)	2,080 (9.4)
Other	1,964 (32.0)	83 (66.4)	1,253 (46.4)	715 (51.1)	1,668 (48.4)	2,760 (52.4)	281 (84.6)	2,178 (78.9)	10,902 (49.2)
No. of asymptomatically carried pathogen exposures									
EAEC	1,448 (23.6)	38 (30.4)	990 (36.7)	474 (33.9)	1,237 (35.9)	1,699 (32.2)	138 (41.6)	813 (29.5)	6,837 (30.9)
*Campylobacter*	1,286 (21.0)	19 (15.2)	381 (14.1)	283 (20.2)	491 (14.3)	1,338 (25.4)	40 (12.0)	547 (19.8)	4,385 (19.8)
ETEC	1,517 (24.7)	10 (8.0)	471 (17.4)	228 (16.3)	635 (18.4)	845 (16.0)	41 (12.3)	629 (22.8)	4,376 (19.7)
aEPEC	834 (13.6)	40 (32.0)	414 (15.3)	274 (19.6)	538 (15.6)	578 (11.0)	74 (22.3)	345 (12.5)	3,097 (14.0)
tEPEC	645 (10.5)	9 (7.2)	275 (10.2)	84 (6.0)	297 (8.6)	528 (10.0)	20 (6.0)	236 (8.6)	2,094 (9.4)
*Shigella*	401 (6.5)	9 (7.2)	169 (6.3)	56 (4.0)	247 (7.2)	282 (5.4)	19 (5.7)	189 (6.9)	1,372 (6.2)

Data are n or n (%). EAEC = enteroaggregative *Escherichia coli.* ETEC = enterotoxigenic *E. coli*. aEPEC = atypical enteropathogenic *E. coli*. tEPEC = typical enteropathogenic *E. coli*.

^*^Children were included if they had 2 complete years of follow-up with qPCR data.

^†^Total linked antibiotic courses are a subset of the total antibiotic courses that could be linked to a diarrheal or surveillance stool sample in the prior 30 d.

^‡^The total number of instances in which a pathogen is exposed to antibiotics. If multiple pathogens are exposed to the same course of antibiotics, each pathogen is counted. Because an antibiotic course could include multiple drug classes, the total by drug class does not equal the total for any antibiotic. The total by subclinical pathogen equals the total for all pathogens.

**Fig. 1.**
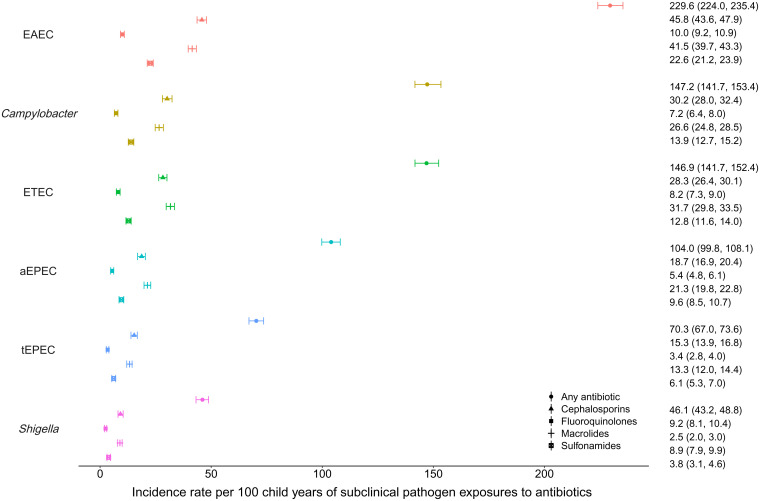
Incidence rates per 100 child-years of asymptomatically carried enteric pathogen exposures to antibiotics among 1,715 children in the MAL-ED cohort.

Overall, for any subclinical bacterial pathogen analyzed, there were 744.1 exposures to antibiotics per 100 child-years (95% CI 729.1 to 760.6) (*SI Appendix*, Table S1). As a subclinical bystander, EAEC was exposed to antibiotics more frequently than any other pathogen, with an incidence rate of 229.6 exposures to any antibiotic per 100 child-years (95% CI 224.0 to 235.4; Fig. 1). The rate of exposure to cephalosporins was 45.8 exposures per 100 child-years (95% CI 43.6 to 47.9), to fluoroquinolones was 10.0 exposures per 100 child-years (95% CI 9.2 to 10.9), to macrolides was 41.5 exposures per 100 child-years (95% CI 39.7 to 43.3), and to sulfonamides was 22.6 exposures per 100 child-years (95% CI 21.2 to 23.9). The other bacterial pathogens were exposed at lower rates overall: 147.2 exposures per 100 child-years (95% CI 141.7 to 153.4) for *Campylobacter*, 146.9 exposures per 100 child-years (95% CI 141.7 to 152.4) for ETEC, 104.0 exposures per 100 child-years (95% CI 99.8 to 108.1) for atypical enteropathogenic *E. coli* (aEPEC), 70.3 exposures per 100 child-years (95% CI 67.0 to 73.6) for typical enteropathogenic *E. coli* (tEPEC), and 46.1 exposures per 100 child-years (95% CI 43.2 to 48.8) for *Shigella.* The results were similar when antibiotic courses were matched to the most recent stool within 7 d (*SI Appendix*, Fig. S1) or 21 d (*SI Appendix*, Fig. S2) instead of 30 d in a sensitivity analysis.

The sites in Bangladesh (1,670.6, 95% CI 1,612.7 to 1,725.3) and Pakistan (1,243.3, 95% CI 1,193.4 to 1,298.0) had the highest incidence rates of subclinical pathogen exposure to antibiotics, while those in Brazil (57.3, 95% CI 47.1 to 66.7) and South Africa (89.4, 95% CI 81.2 to 97.6) had the lowest incidence rates (*SI Appendix*, Table S1). Overall, macrolides and cephalosporins were the most frequently used antibiotics for all indications, with 149.3 (95% CI 137.6 to 160.7) and 145.9 (95% CI 137.5 to 154.6) courses per 100 child-years, respectively (*SI Appendix*, Table S2). Macrolides were overwhelmingly used in the Bangladesh and Peru sites and cephalosporins in the Pakistan, Bangladesh, and India sites. The median duration of antibiotic courses was 5 d (interquartile range [IQR] 3 to 7 d), such that the total days of antibiotic exposure per 100 child-years for each subclinical pathogen was approximately 5 times the incidence rate (*SI Appendix*, Fig. S3).

**Fig. 2.**
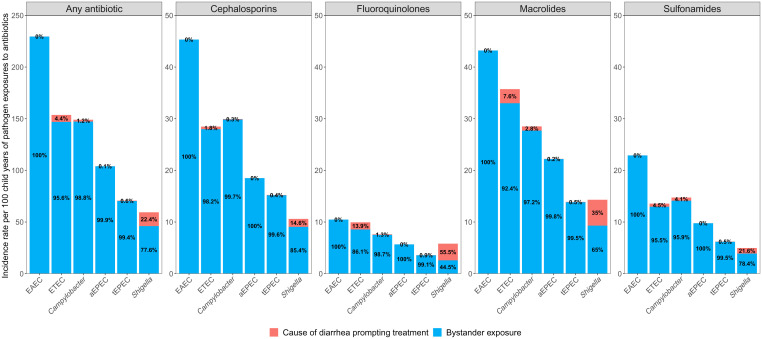
Incidence rates per 100 child-years of enteric pathogen exposures to antibiotics and the proportion of exposures that were due to diarrheal prompting treatment versus bystander exposure among 1,715 children in the MAL-ED cohort.

Nearly all antibiotic exposures for *Campylobacter* (98.8%), ETEC (95.6%), and tEPEC (99.4%), and the majority for *Shigella* (77.6%) occurred when they were subclinical infections and not when they were the cause of treatment (Fig. 2). When examined by drug class, all four pathogens had a higher proportion of antibiotic exposures to cephalosporins, fluoroquinolones, macrolides, and sulfonamides as subclinical infections, except for *Shigella*, when exposed to fluoroquinolones (44.5% subclinical exposure versus 55.5% diarrhea treatment). A sensitivity analysis, in which antibiotic courses were matched to the nearest stool within 21 d, instead of 30 d, yielded similar results (*SI Appendix*, Fig. S4). In an additional sensitivity analysis in which diarrhea etiology was more liberally assigned if the pathogen was detected at any quantity (quantification cycle [Cq] <35) during diarrhea, the percentage of antibiotic exposure that occurred when the pathogen was the cause of diarrhea increased. However, the majority of exposures still occurred during subclinical infections: *Campylobacter* (86.0%), ETEC (77.8%), tEPEC (80.9%), and *Shigella* (68.1%) (*SI Appendix*, Fig. S5).

**Fig. 3.**
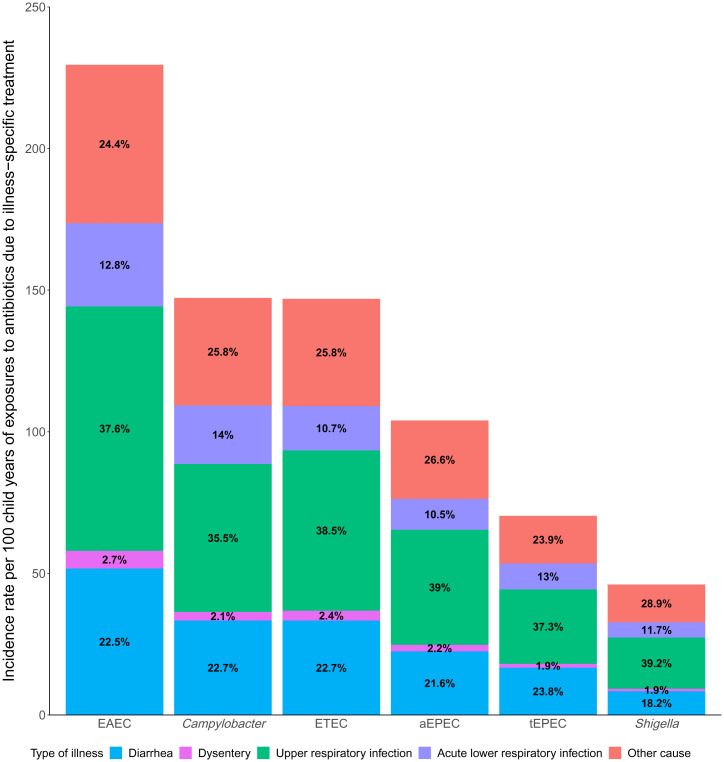
Incidence rates per 100 child-years of enteric pathogen exposures to antibiotics and the proportion of exposures due to type of illness among 1,715 children in the MAL-ED cohort.

Together, upper respiratory infections (URIs) (37.6%) and acute lower respiratory infections (ALRIs) (12.3%) accounted for half (49.9%) of antibiotic courses in which bystander pathogens were exposed (Fig. 3 and *SI Appendix*, Table S3). Bystander pathogen exposure to antibiotics due to diarrheal and dysentery illnesses combined only accounted for approximately one-fourth (24.6%) of antibiotic exposures (with dysentery accounting for <3% of illness for each bystander pathogen). When subset to bystander pathogen exposures to fluoroquinolones and macrolides only, URIs and ALRIs were still attributed as the reason for antibiotic use in nearly half (45.5%) of all antibiotic courses; however, the proportion of diarrheal and dysentery illnesses as the reason for antibiotic use increased to more than one-third (40.7%) (*SI Appendix*, Fig. S6). Upon further examination of fluoroquinolone and macrolide drug classes individually, the incidence rates of exposure due to URIs and diarrheal illnesses were nearly equivalent across bystander pathogens (*SI Appendix*, Table S4).

Certain childhood characteristics were associated with lower rates of bystander exposure to antibiotics, which may be due to either less frequent antibiotic receipt or carriage of pathogens or both (*SI Appendix*, Table S5). For every 0.5-U increase in WAMI (access to improved water/sanitation, assets, maternal education, and income) score, a measure of socioeconomic status, there were 10% fewer total exposures of subclinical bacterial pathogens to antibiotics (incidence rate ratio [IRR] 0.90, 95% CI 0.86 to 0.94). In addition, female sex (IRR 0.87, 95% CI 0.84 to 0.89), exclusive breastfeeding (IRR 0.98, 95% CI 0.97 to 0.99), treated water (IRR 0.94, 95% CI 0.90 to 0.98), and access to an improved latrine (IRR 0.92, 95% CI 0.89 to 0.96) were associated with fewer total exposures of subclinical bacterial pathogens to antibiotics. However, there was no association with WAMI score (IRR 1.04, 95% CI 0.94 to 1.15) and treated water (IRR 0.96, 95% CI 0.90 to 1.02), and the associations with improved source of drinking water (IRR 0.78, 95% CI 0.64 to 0.94) and access to improved latrine (IRR 0.84, 95% CI 0.76 to 0.93) strengthened when subset to exposures to fluoroquinolones or macrolides.

**Fig. 4.**
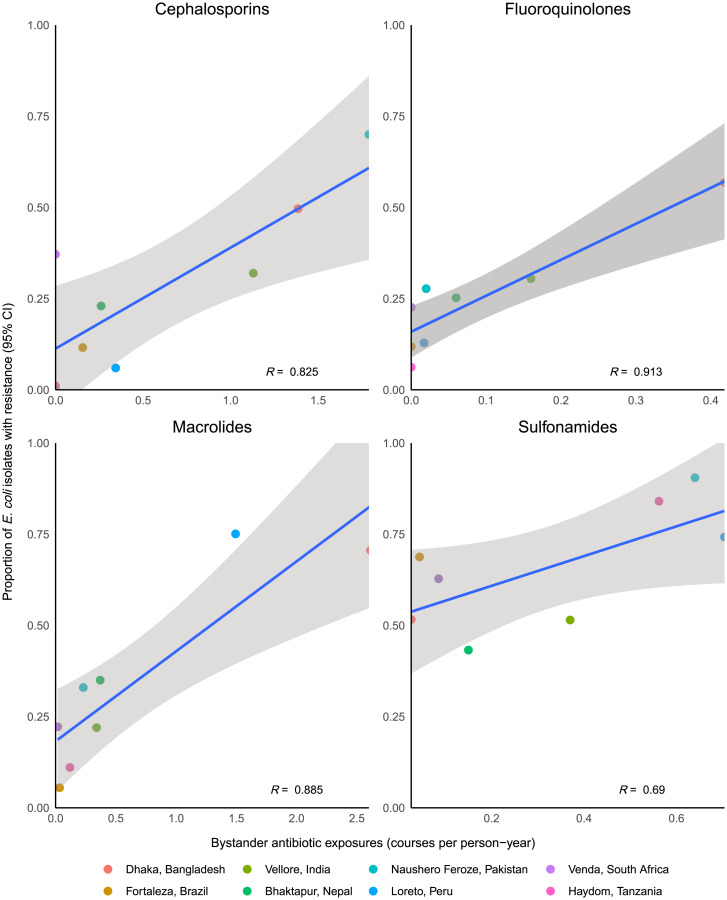
Associations between the incidence of bystander antibiotic exposures and the prevalence of antibiotic resistance in *E. coli* isolates at each of the 8 sites of the MAL-ED study. Analysis includes 153 isolates from Bangladesh, 362 isolates from Brazil, 400 isolates from India, 400 isolates from Nepal, 400 isolates from Pakistan, 311 isolates from Peru, 234 isolates from South Africa, and 370 isolates from Tanzania. *Top Left*: exposure to cephalosporins and resistance to ceftriaxone; *Top Right*: exposure to fluoroquinolones and resistance to ciprofloxacin; *Bottom Left*: exposure to macrolides and resistance to azithromycin; *Bottom Right*: exposure to sulfonamides and resistance to trimethoprim/sulfamethoxazole. R denotes the Pearson correlation coefficient.

A total of 2,630 *E. coli* isolates from 505 children had antibiotic susceptibility data, and the prevalence of resistance varied by site and drug class (Fig. 4). A total of 87% of isolates exhibited resistance to at least one antibiotic. Bystander exposure to macrolides in the past 30 d was associated with a 29% (95% CI 13 to 47) increase in the prevalence of macrolide resistance in cultured *E. coli* isolates (*SI Appendix*, Table S6). Recent bystander exposures to cephalosporins, fluoroquinolones, and sulfonamides were associated with smaller (<10%), nonsignificant increases in resistance to the corresponding drug class. Conversely, the incidence of class-specific antibiotic exposures at the site level were strongly correlated with the prevalence of class-specific resistance among all isolates from that site, particularly for fluoroquinolones (*R =* 0.91) and macrolides (*R* = 0.89; Fig. 4). Macrolide exposure and prevalence of macrolide resistance were highest in the Bangladesh and Peru sites, while fluoroquinolone exposure and prevalence of fluoroquinolone resistance were highest in the Bangladesh and India sites.

## Discussion

The concurrent high prevalence of subclinical enteric infections and frequent antibiotic use among young children in low-resource settings resulted in strikingly high rates of bystander exposure to antibiotics for bacterial enteropathogens, including more than 10 exposures per year in some sites. The frequency of exposure was driven largely by the prevalence of each pathogen; for example, EAEC was detected in more than half of the nondiarrheal stools in MAL-ED (5) and accounted for the largest share of bystander exposure events. The rates of bystander exposure varied substantially by site, with higher rates in the South Asian sites compared to the sub-Saharan Africa and South American sites. These trends align with a previous analysis of antibiotic use overall in this study (9), and can be explained by higher rates of illness (5, 9), more frequent antibiotic treatment of illnesses (9), and higher prevalence of asymptomatic infections (5) in the South Asian sites. Variation in treatment frequency is likely due to differences in treatment practices and the availability of antibiotics across regions related to prescription requirements and drug shortages (13–16).

For the bacterial pathogens studied, almost all of the antibiotic exposure occurred when the pathogens were not the target of treatment. Even when diarrhea etiology was liberally defined by the detection of a pathogen at any quantity during diarrhea, the majority of antibiotic exposures occurred outside of diarrhea episodes. Therefore, interventions to reduce selection pressure on pathogens that are of particular concern for AMR, such as *Shigella* and *Campylobacter*, must account for exposures that occur during subclinical infections.

*Shigella* was the only pathogen for which treatment of shigellosis accounted for a substantial proportion of total antibiotic exposures (i.e., *Shigella* was the target pathogen), although such treatment accounted for only 22% of exposures of *Shigella* to antibiotics. *Shigella* is the leading cause of dysentery (17) for which antibiotics are indicated (11), and was the leading cause of antibiotic use compared to other diarrhea etiologies among children in MAL-ED (10). However, the majority of exposures to cephalosporins and macrolides, drug classes of concern for resistant shigellosis, still occurred when *Shigella* was not the cause of treatment.

Interestingly, the predictors of bystander exposure differed from the predictors of antibiotic use overall, documented previously (9). Specifically, while higher socioeconomic status was associated with an increase in antibiotic use overall, it was associated with fewer bystander exposures. Furthermore, exclusive breastfeeding and improved access to water and sanitation were protective for bystander exposures, but were not associated with antibiotic use overall. This is likely due to fewer asymptomatic infections among children with these characteristics (6, 8, 18) and suggests that while antibiotic use may be more frequent in high socioeconomic status populations, cumulative antibiotic exposure for enteric pathogens may be driven by lower socioeconomic status populations.

These results highlight the potential ancillary benefits of antibiotic stewardship interventions for reducing selection on bystander pathogens. An analysis of diverse data sources in the United States suggested that preventing inappropriate antibiotic use could prevent up to almost half of all antibiotic exposures for some pathogen and drug combinations (19). Antibiotics are frequently inappropriately used to treat viral causes of diarrhea (10), and improved stewardship would reduce exposure for bacterial enteropathogens and limit collateral effects on the microbiota. However, because point-of-care diagnostics are largely unavailable and clinical treatment algorithms for determining which diarrhea episodes would respond to treatment are inadequate, efforts to reduce antibiotic prescribing for viral diarrhea have had limited success. Interventions that prevent the indicating illness from occurring, such as vaccines, may be more successful at reducing antibiotic use for specific diarrhea etiologies. For example, rotavirus vaccines were estimated to prevent 13.6 million antibiotic-treated diarrhea episodes each year among children in the first 2 y of life in low- and middle-income countries (20). The protective associations observed in this study for improved sanitation and treated drinking water with bystander antibiotic exposure suggest that water, sanitation, and hygiene interventions may also reduce this burden.

While antibiotic susceptibility testing was not possible for the enteropathogens studied due to the application of molecular diagnostics, the implicit assumption underlying this work is that antibiotic exposure contributes to resistance in the pathogens studied. The association between antibiotic exposures and resistance in the *E. coli* isolates informs the relevance of bystander exposures. While the associations between recent antibiotic exposure and resistance at the individual level were generally small and inconsistent across drug classes, the site-level ecological associations between exposure and resistance were strong, suggesting the effects of bystander exposure are better captured at the community level. Previous work has demonstrated associations between antibiotic use and carriage of resistant organisms at levels spanning the individual (e.g., (21–23)), community (24–26), and country (27, 28), both over time (29, 30) and across diverse geographies. Where analyzed, resistance has been linked to treatment failures necessitating the use of second-line therapies (22, 31, 32) as well as adverse long-term outcomes and sequelae (33, 34). Detailed characterization of enteropathogen carriage and diarrheal etiology in the small but richly sampled MAL-ED cohort provided an opportunity to quantify bystander antibiotic exposure and resistance from direct measurement in this study. However, alternative designs—in particular, studies enrolling larger cohorts—are needed to document the implications of such exposures for rarer downstream health outcomes, including treatment failure and adverse clinical sequelae. Such studies remain important for measurement of the health burden of AMR and for defining the value proposition of vaccination and stewardship interventions that may reduce the frequency of bystander antibiotic exposure (35, 36).

The primary strength of this study was the collection of relevant pathogen infection and antibiotic use data from a longitudinal cohort, such that minimal assumptions to connect diverse data sources were required, unlike a prior study (3). This study was limited by the identification of subclinical infections from stools collected within the previous 30 d. Pathogen prevalence at this time may under- or overestimate the pathogens present during antibiotic treatment, especially since prevalence increased with age for many pathogens (5). However, sensitivity analyses using different time windows yielded similar results. The majority of antibiotic courses had a stool collected within 30 d due to monthly sampling of nondiarrheal stools, such that extrapolation to courses that could not be linked to a stool sample was limited within a 30-d period. In addition, we inferred the indications for antibiotic treatment based on the symptoms reported at the time of treatment in lieu of a clinician’s diagnosis. Finally, the generalizability of these results may be limited given the restriction to eight sites. Antibiotic use patterns varied by site and may have changed since the time of the study in response to local resistance patterns, such that the incidence rates may not be applicable to other settings. Future work to transport results from this study to a broader range of settings using more representative survey datasets would help define the global importance of bystander exposure. However, the evidence for the high relative frequency of bystander exposure is likely to be consistent across settings and time periods.

In summary, estimates of the frequency and impact of antibiotic exposure that are limited to the target pathogen of treatment largely underestimate the total selection pressure to enteric pathogens. Pathogens that are not common causes of diarrhea in these settings, such as EAEC, ultimately experience the highest frequency of exposure to antibiotics due to frequent subclinical carriage at the time of treatment. This work expands the value proposition for antibiotic stewardship programs and informs which types should be prioritized. Specifically, for example, our results suggest that reducing antibiotic use for respiratory infections may have a larger impact on the development of resistance in enteric pathogens than reducing antibiotic use for the treatment of enteric bacteria directly. The temporal intersection of enteric infections and antibiotic use among children in low-resource settings results in a population in which antibiotic stewardship interventions could have an outsized impact due to the high frequency of bystander exposure.

## Materials and Methods

### Study Design and Participants.

The MAL-ED study design has been previously detailed (37). The study was conducted from November 2009 through February 2014 at eight sites: Dhaka, Bangladesh; Fortaleza, Brazil; Vellore, India; Bhaktapur, Nepal; Loreto, Peru; Naushero Feroze, Pakistan; Venda, South Africa; and Haydom, Tanzania. Children were enrolled from birth (younger than 17 d of age) and were followed for 2 y. All of the sites received ethical approval from their respective governmental, local institutional, and collaborating institutional review boards. Written informed consent was obtained from the parent or guardian of each child.

### Surveillance and Sample Collection.

Twice per week, fieldworkers conducted home visits to collect information on sociodemographics, antibiotic use, presence of illness, and feeding practices. The WAMI index was used as a marker of socioeconomic status. Diarrhea was defined as three or more loose stools in a 24-h period, and dysentery was defined as at least one stool with visible blood, both by caregiver report. URI was defined previously (9) as cough or shortness of breath by caregiver report, and ALRI was defined as cough or shortness of breath with an average respiratory rate greater than age-specific cutoffs for a rapid rate from two measurements by fieldworkers (38). Distinct illness episodes were separated by 2 d free of the defined illness.

Any antibiotic use and use of specific antibiotic drug classes were reported by caregivers for every day of follow-up. In this analysis, we focused on any antibiotic use (i.e., all drug classes) and the use of cephalosporins, fluoroquinolones, macrolides, and sulfonamides, specifically, which are used to treat bacterial diarrhea and are relevant for the development of resistance (11, 39). Drug classes in the “other” category included penicillins, tetracyclines, metronidazole, and unknown/other. Distinct antibiotic courses were defined if separated by at least two antibiotic-free days, as previously outlined (9). Nondiarrheal stool samples were collected every month within ±7 d of the child’s birthday, and additional stool samples were collected during each diarrhea episode. Sociodemographic characteristics were collected every 6 mo, and averaged values across time points were included in the analysis.

### Stool Testing.

The QIAamp Fast DNA Stool Mini Kit (Qiagen) was used to extract total nucleic acid from stool samples, described elsewhere (40). Quantitative PCR (qPCR) using AgPath One Step real-time PCR kit (Thermo Fisher) via the TaqMan Array Card platform was used to detect 29 enteropathogens (17). We used extrinsic controls (bacteriophage MS2 and phocine herpesvirus) to monitor extraction and amplification and extraction blank to exclude laboratory contamination. Positive detection was defined at a Cq threshold <35. Bacterial pathogens that were present in at least 5% of nondiarrheal stools were included in these analyses: *Campylobacter* spp., *Shigella*, enterotoxigenic *E. coli* (ETEC), tEPEC, aEPEC, and EAEC.

Five indole-positive, lactose-fermenting colonies (i.e., *E. coli)* from all of the stool samples were cultured, pooled, and stored at −80 °C. We randomly selected 50 children from each site who had *E. coli* isolates cultured from nondiarrheal stools collected at 3, 6, 9, 12, 15, 18, 21, and 24 mo of age and conducted antibiotic susceptibility testing on those isolates. Because a subset of pooled *E. coli* isolates was archived at the Peru site, fewer than 8 isolates from more than 50 children (*n* = 157) were included. Antibiotic susceptibility testing was performed using the E-test for azithromycin and disk diffusion for all of the other antibiotics. Susceptibility cutoffs were derived from the literature for azithromycin. For all other antibiotics, susceptibility cutoffs were defined by the Clinical Laboratory Standards Institute Guidelines (*SI Appendix*, Table S7). Ceftriaxone, ciprofloxacin, azithromycin, and trimethoprim-sulfamethoxazole resistance were used as indicators of class-specific resistance to cephalosporins, fluoroquinolones, macrolides, and sulfonamides, respectively.

### Statistical Analysis.

To identify the pathogens that were carried asymptomatically at the time of antibiotic treatment, we linked each antibiotic course to the nearest nondiarrheal or diarrheal stool sample collected within 30 d before starting the antibiotic course. Any pathogen detected in that stool was assumed to have been exposed to the antibiotic while present asymptomatically (i.e., a bystander exposure event occurred), unless that pathogen was the target pathogen of treatment (i.e., the cause of diarrhea prompting treatment, defined below). For courses in which a stool sample with valid pathogen data were not available in the past 30 d, we extrapolated from courses in which data were available by site and drug class for class-specific analyses. In sensitivity analyses, we restricted ourselves to courses with samples collected within 21 d before starting the antibiotic course based on an analysis of the average duration of pathogen carriage after a diarrheal episode (41) and also within 7 d before starting the antibiotic course.

Indicating illnesses for antibiotic treatment were determined by the overlap between antibiotic use and reported symptoms, as previously outlined (9, 10). If an antibiotic course overlapped on any day with an ALRI, then we considered the course to be attributed to ALRI. If the course was not due to ALRI but overlapped with an episode of dysentery, then the course was attributed to dysentery. If the course was not attributed to ALRI or dysentery but overlapped on any day with diarrhea, then we considered diarrhea to be the indication for treatment. Finally, if the course overlapped with symptoms of a URI but not the conditions above, the course was attributed to a URI.

Target pathogens of antibiotic use for diarrhea were determined by attributing etiology to diarrheal episodes that were identified as responsible for antibiotic treatment. If multiple diarrhea episodes occurred during an antibiotic course, then we used pathogen data from the first diarrheal episode to define etiology. To determine which specific enteric pathogens caused the antibiotic-treated diarrhea, we constructed mixed-effects models using diarrheal and nondiarrheal stools that associated pathogen quantity with diarrhea to calculate pathogen-specific attributable fractions for each episode (AFe), adjusting for other pathogens, age, sex, test batch, site, and individual. Etiology-specific incidence estimates for all diarrhea episodes using these methods in comparison with pathogen prevalence during diarrhea have been published previously (17). We included the top 10 causes of diarrhea in MAL-ED: adenovirus 40/41, astrovirus, *Campylobacter jenjui/Campylobacter coli*, *Cryptosporidium*, norovirus, rotavirus, sapovirus, *Shigella*, heat-stable enterotoxigenic *E. coli* (ST-ETEC), and tEPEC. A pathogen was presumed to be the causative agent of diarrhea if the AFe was >0.5. For antibiotic courses in which a pathogen was identified as the cause of diarrhea prompting treatment, the antibiotic course was not considered a cause of bystander exposure to that pathogen, even if it was also detected in the stool collected in the prior 30 d. In a sensitivity analysis, we considered a pathogen to be the causative agent of diarrhea if detected at any quantity (Ct < 35).

To quantify bystander exposure or exposures of subclinical infections to antibiotics, we calculated the incidence rate per 100 child-years as the number of subclinical pathogen exposures divided by the observed person-time, divided by the proportion of antibiotic courses that could be linked to a stool within the prior 30 d (to extrapolate to all courses). We also calculated the total number of days of antibiotic exposure per 100 child-years for each subclinical pathogen by summing the duration of each course. These rates were calculated for each bacterial pathogen of interest and for any bacterial pathogen. For the latter, if multiple subclinical pathogens were exposed to the same course of antibiotics, then the exposure was counted for each pathogen.

We then calculated the proportion of antibiotic exposures for each pathogen that occurred as bystander exposure (i.e., when the pathogen was not considered to be the cause of antibiotic treatment) and the proportion of antibiotic exposures that occurred when the pathogen was the cause of treatment (i.e., caused the diarrhea prompting treatment).

We quantified the incidence per 100 child-years and proportion of bystander exposure events that were attributed to the treatment of diarrhea, dysentery, URI, ALRI, and other causes for all courses of antibiotics, fluoroquinolone, and macrolide courses specifically.

Finally, we estimated the associations between child characteristics and the frequency of bystander exposure to characterize the population in which this exposure occurs. We used Poisson regression to estimate IRRs for the total number of times that bacterial subclinical pathogens were exposed to any antibiotic. Separately, we restricted the outcome to instances in which bacterial subclinical pathogens were exposed to fluoroquinolones or macrolides. In both instances, we ran individual models assessing each child characteristic alone while adjusting for site and multivariable models that included all child characteristics and site.

All of the analyses were performed overall, at each site, and for specific pathogens and drug classes. We estimated the 95% CIs for all of the estimates except those from the Poisson models by bootstrap with 1,000 resamples. Wald intervals were used for the Poisson models.

To associate bystander antibiotic exposure with antibiotic resistance at the individual level, we estimated the effect of class-specific antibiotic exposure in the last 30 d on the prevalence of resistance to the same drug class in the cultured *E. coli* isolates. Because *E. coli* are ubiquitously present in stool, commensal, and therefore not the target of antibiotic treatment, all of the observed antibiotic exposures were considered bystander exposures to *E. coli*. To estimate risk ratios, we used the Poisson approximation for log-binomial regression with generalized estimating equations to account for multiple isolates cultured from the same child, adjusting for site, age, sex, WAMI index, and hospitalization in the last 90 d. To compare rates of bystander exposures with antibiotic resistance at the community level, we associated site-level incidence of class-specific antibiotic exposures with the prevalence of class-specific resistance among all of the isolates from that site using linear regression, and we estimated the correlation between these two variables using the Pearson correlation coefficient (*R*). All of the statistical analyses were performed via R software, version 4.0.2 (R Foundation for Statistical Computing).

### Ethics Approvals and Data Availability.

This study involves human participants. For the parent study, ethical approval was obtained from the institutional review boards at the University of Virginia School of Medicine (Charlottesville, VA) (14595), and at each of the participating research sites: Ethical Review Committee, International Centre for Diarrhoeal Disease Research, Bangladesh (Bangladesh); Committee for Ethics in Research, Universidade Federal do Ceara, and National Ethical Research Committee, Health Ministry, Council of National Health (Brazil); Institutional Review Board, Christian Medical College, Vellore, and Health Ministry Screening Committee, Indian Council of Medical Research (India); Institutional Review Board, Institute of Medicine, Tribhuvan University, Ethical Review Board, Nepal Health Research Council, and Institutional Review Board, Walter Reed Army Institute of Research (Nepal); Institutional Review Board, Johns Hopkins University, and PRISMA Ethics Committee; Health Ministry, Loreto (Peru); Ethical Review Committee, Aga Khan University (Pakistan); Health, Safety and Research Ethics Committee, University of Venda, and Department of Health and Social Development, Limpopo Provincial Government (South Africa); and Medical Research Coordinating Committee, National Institute for Medical Research, and Chief Medical Officer, Ministry of Health and Social Welfare (Tanzania). For the present study, we obtained ethical approval at the University of Virginia School of Medicine (Charlottesville, VA) (22398) and Emory University (Atlanta, GA) (STUDY00003285). Participants gave informed consent to participate in the study before taking part. The statistical analysis plan is available at https://osf.io/3asxh. Deidentified participant data from the MAL-ED study is publicly available at ClinEpiDB.org after approval of a proposal by the study’s principal investigators (PIs).

## Supplementary Material

Supplementary File

## Data Availability

Some study data available (deidentified participant data from the MAL-ED study is publicly available at ClinEpiDB.org after approval of a proposal by the study PIs).
