# Supplementary material for: Frequency of bystander exposure to antibiotics for enteropathogenic bacteria among young children in low-resource settings
Source: Proc Natl Acad Sci U S A. 2022 Aug 29;119(36):e2208972119. doi: 10.1073/pnas.2208972119 (PMC9457395; doi:10.1073/pnas.2208972119)
Supplement: Supplementary File [file pnas.2208972119.sapp.pdf]

Supplementary appendix

Frequency of bystander exposure to antibiotics for enteropathogenic bacteria among young children in low-resource settings

Elizabeth T Rogawski McQuade, Stephanie A Brennhofer, Sarah E Elwood, Timothy L McMurry, Joseph A Lewnard, Estomih R Mduma, Sanjaya Shrestha, Najeeha Iqbal, Pascal O Bessong, Gagandeep Kang, Margaret Kosek, Aldo A M Lima, Tahmeed Ahmed, Jie Liu, Eric R Houpt, James A Platts-Mills

Correspondence to: Elizabeth T Rogawski McQuade
Email: erogaws@emory.edu

Table S1. Site-specific incidence rates of asymptomatically carried pathogen exposures to antibiotics for the most common bacterial infections among 1715 children in the MAL-ED cohort.

Asymptomatically carried pathogens	Dhaka, Bangladesh	Fortaleza, Brazil	Vellore, India	Bhaktapur, Nepal	Loreto, Peru	Naushero Feroze, Pakistan	Venda, South Africa	Haydom, Tanzania	Overall
EAEC	394.6 (375.2, 413.4)	17.4 (11.7, 23.1)	246.9 (235.9, 258.1)	113.6 (105.8, 122.3)	343.2 (329.2, 356.5)	400.8 (381.6, 422.4)	37.1 (32.1, 42.4)	243.4 (234.6, 253.7)	229.6 (224.0, 235.4)
Campylobacter	350.4 (327.8, 372.7)	8.7 (4.8, 13.0)	95.0 (83.2, 107.0)	67.8 (59.7, 75.9)	136.2 (122.9, 150.4)	315.7 (292, 341.1)	10.8 (7.7, 14.2)	163.8 (152.6, 176.2)	147.2 (141.7, 153.4)
ETEC	413.4 (394.1, 432.7)	4.6 (1.6, 7.7)	117.4 (107.6, 126.8)	54.6 (47.1, 61.9)	176.2 (163.0, 189.8)	199.4 (184.5, 215.4)	11.0 (7.9, 14.6)	188.3 (176.2, 200.7)	146.9 (141.7, 152.4)
aEPEC	227.3 (212.3, 242.3)	18.3 (13.9, 22.8)	103.2 (92.5, 114.0)	65.7 (58.4, 73.7)	149.3 (136.4, 162.3)	136.4 (122.7, 148.7)	19.9 (16.3, 23.6)	103.3 (93.9, 113.5)	104.0 (99.8, 108.1)
tEPEC	175.8 (161.2, 190.6)	4.1 (1.7, 6.7)	68.6 (60.9, 76.9)	20.1 (15.6, 24.9)	82.4 (73.0, 92.4)	124.6 (111.5, 136.9)	5.4 (3.4, 7.6)	70.7 (61.7, 79.6)	70.3 (67.0, 73.6)
Shigella	109.3 (97.8, 122.1)	4.1 (1.7, 6.6)	42.1 (34.9, 50.0)	13.4 (9.8, 17.9)	68.5 (58.9, 79.1)	66.5 (56.7, 76.8)	5.1 (3.0, 7.3)	56.6 (49.1, 64.1)	46.1 (43.2, 48.8)
Any bacterial pathogen	1670.6 (1612.7, 1725.3)	57.3 (47.1, 66.7)	673.3 (645.8, 700.5)	335.3 (317.6, 354.4)	955.9 (917.8, 994.7)	1243.3 (1193.4, 1298.0)	89.4 (81.2, 97.6)	826 (797.9, 855.0)	744.1 (729.1, 760.6)

Data are incidence rates per 100 child years with 95% confidence intervals (CIs). EAEC = enteroaggregative *Escherichia coli.* ETEC= enterotoxigenic *Escherichia coli*. aEPEC = atypical enteropathogenic *Escherichia coli*. tEPEC = typical enteropathogenic *Escherichia coli*.

Figure S1. Sensitivity analysis: Incidence rates per 100 child years of asymptomatically carried enteric pathogen exposures to antibiotics among 1715 children in the MAL-ED cohort when bystander pathogens were identified from the nearest stool collected in the 7 days prior to the antibiotic course.

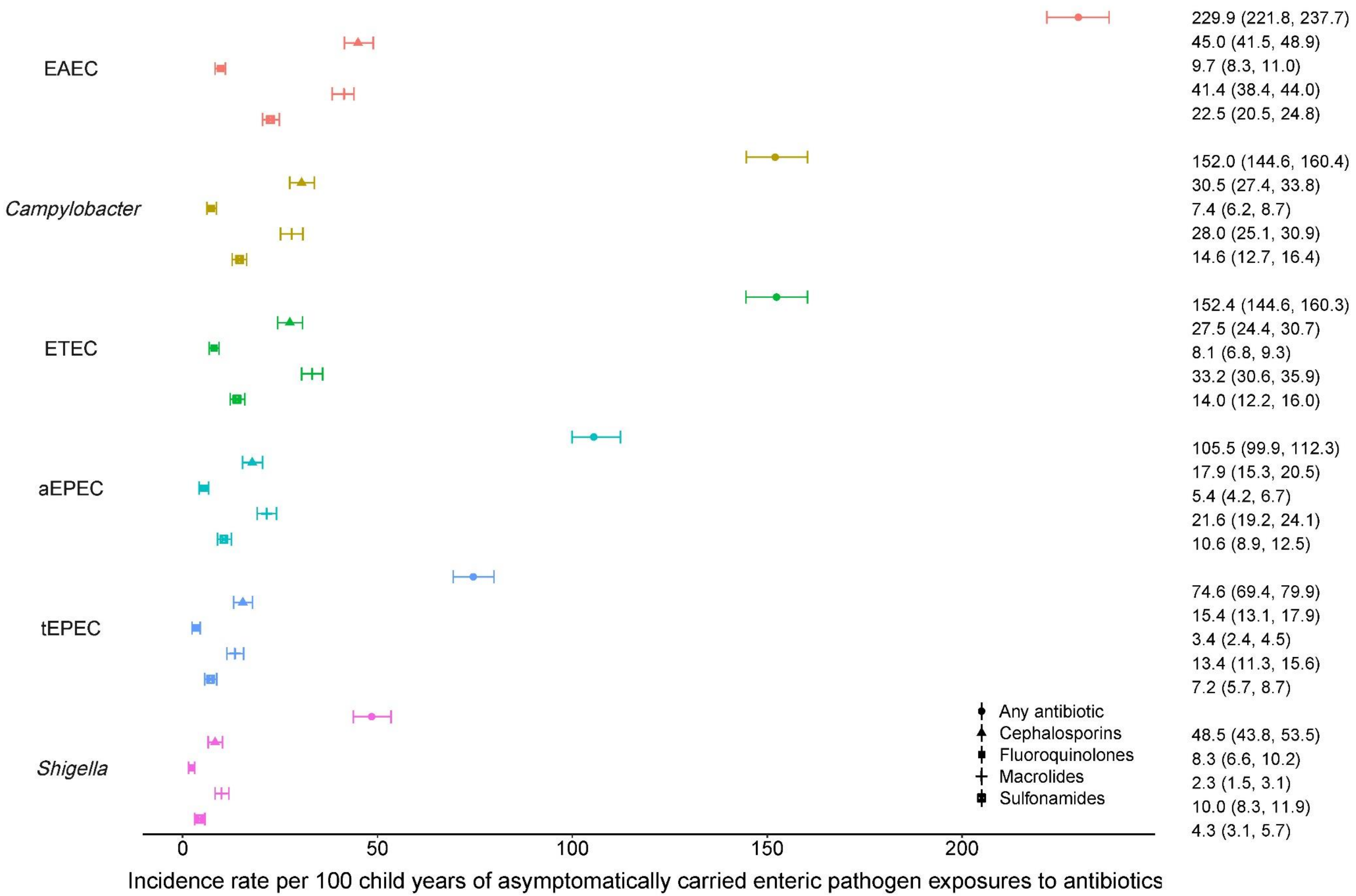

Figure S2. Sensitivity analysis: Incidence rates per 100 child years of asymptomatically carried enteric pathogen exposures to antibiotics among 1715 children in the MAL-ED cohort when bystander pathogens were identified from the nearest stool collected in the 21 days prior to the antibiotic course.

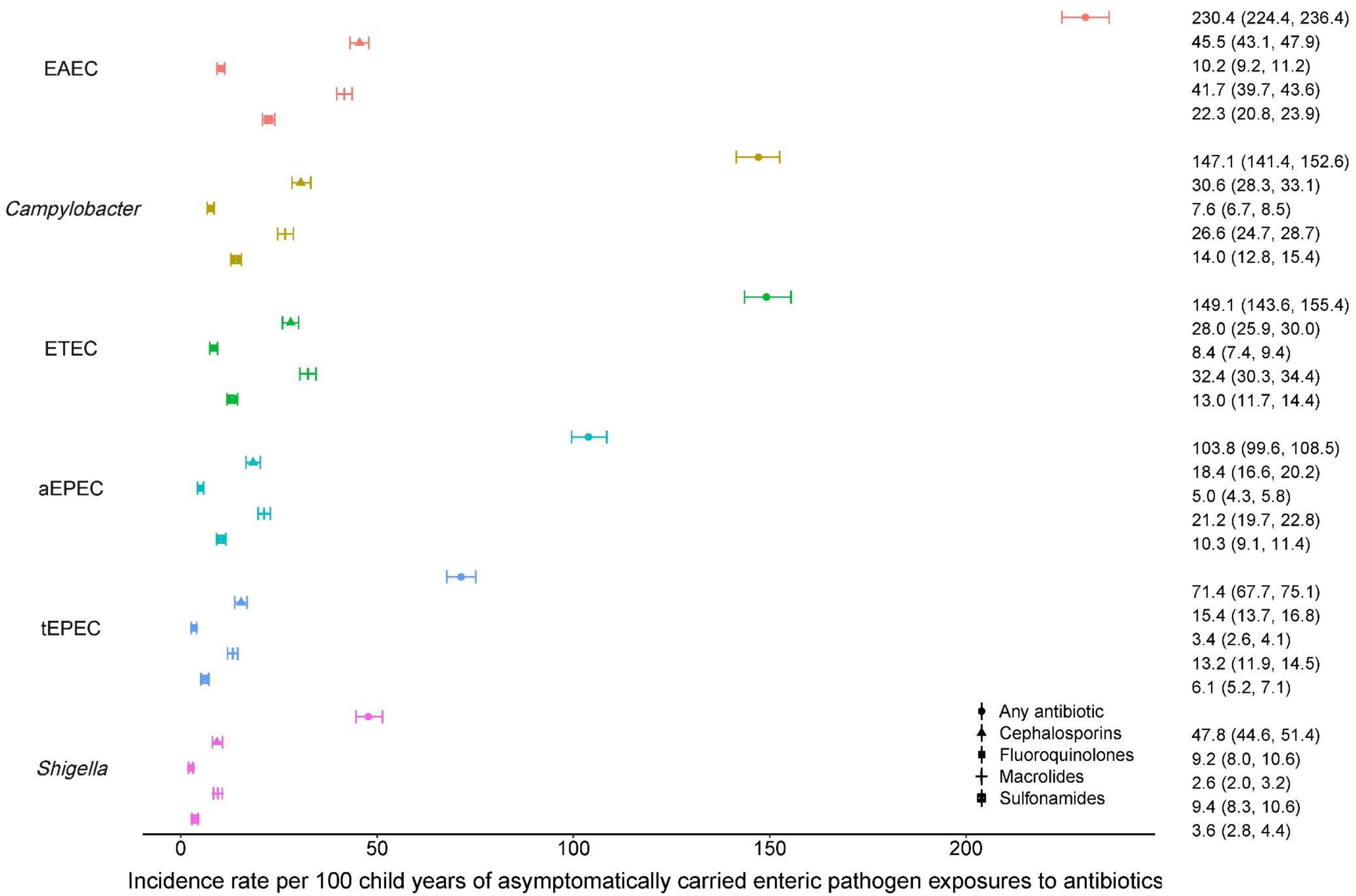

Table S2. Site and drug class-specific incidence rates of asymptomatically carried pathogen exposures to antibiotics for any bacterial pathogen among 1715 children in the MAL-ED cohort.

Drug classes	Dhaka, Bangladesh	Fortaleza, Brazil	Vellore, India	Bhaktapur, Nepal	Loreto, Peru	Naushero Feroze, Pakistan	Venda, South Africa	Haydom, Tanzania	Overall
Cephalosporins	367.0 (330.8, 401.2)	14.2 (8.2, 20.5)	211.7 (188.3, 237.7)	52.5 (41.3, 64.6)	29.4 (19.3, 39.9)	419.9 (383.7, 458.2)	0.5 (0.0, 1.4)	3.3 (0.3, 7.4)	145.9 (137.5, 154.6)
Fluoroquinolones	169.2 (144.0, 193.7)	0.0 (0.0, 0.0)	39.6 (29.8, 50.6)	19.2 (12.6, 26.6)	43.3 (29.3, 61.0)	27.8 (20.8, 36.3)	0.8 (0.0, 2.8)	1.8 (0.0, 4.8)	38.4 (33.9, 43.1)
Macrolides	689.9 (651.4, 730.3)	2.3 (0.4, 4.9)	56.4 (43.3, 69.5)	66.1 (53.2, 79.0)	277.8 (248.5, 306.7)	61.8 (46.9, 75.9)	5.7 (2.5, 9.2)	36.5 (23.5, 50.4)	149.3 (137.6, 160.7)
Sulfonamides	7.6 (3.1, 12.9)	2.8 (0.0, 6.5)	65.3 (49.7, 83.1)	32.8 (24.8, 41.4)	170.9 (143.8, 197.3)	131.2 (112.8, 150.9)	7.3 (4.1, 11.1)	134.1 (113.2, 157)	69.8 (63.9, 76.8)
Other	535.2 (493.1, 580.4)	38.1 (29.0, 47.4)	312.4 (282.4, 342.7)	171.3 (155.0, 187.5)	462.8 (430.4, 497.1)	651.1 (600.3, 701.1)	75.6 (68.1, 83.2)	652.1 (619.8, 684.5)	366.1 (352.8, 380.2)

Data are incidence rates for any bacterial pathogen per 100 child years with 95% confidence intervals (CIs).

Figure S3. Days of antibiotic exposure per 100 child years for asymptomatically carried enteric pathogens among 1715 children in the MAL-ED cohort.

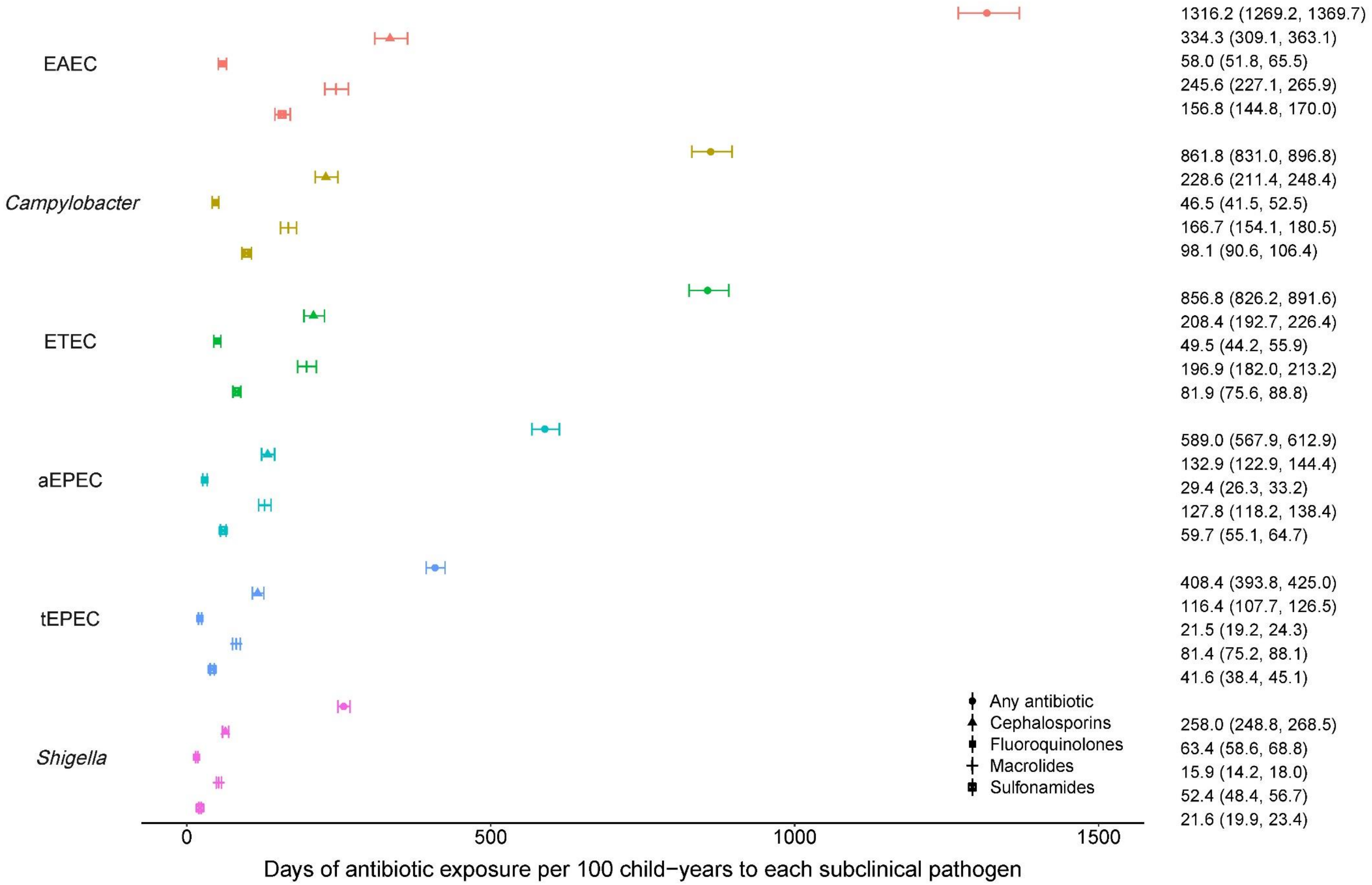

Figure S4. Sensitivity analysis: Incident rates per 100 child years of enteric pathogen exposures to antibiotics and the proportion of exposures that were due to diarrheal prompting treatment vs. bystander exposure among 1715 children in the MAL-ED cohort when bystander pathogens were identified from the nearest stool collected in the 21 days prior to the antibiotic course.

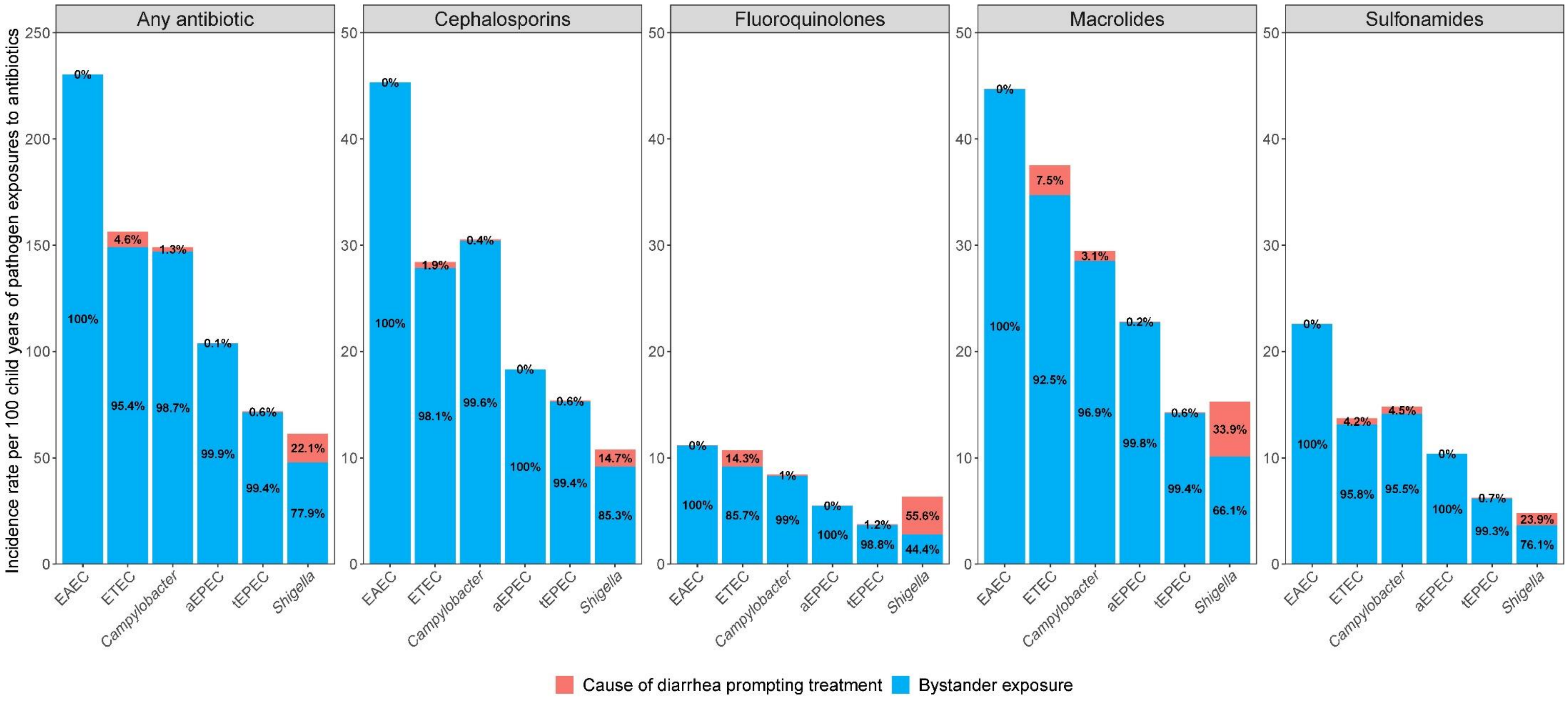

Figure S5. Sensitivity analysis: Incident rates per 100 child years of enteric pathogen exposures to antibiotics and the proportion of exposures that were due to diarrheal prompting treatment vs. bystander exposure among 1715 children in the MAL-ED cohort when diarrhea etiology was defined by the detection of a pathogen by qPCR at any quantity (quantification cycle threshold < 35).

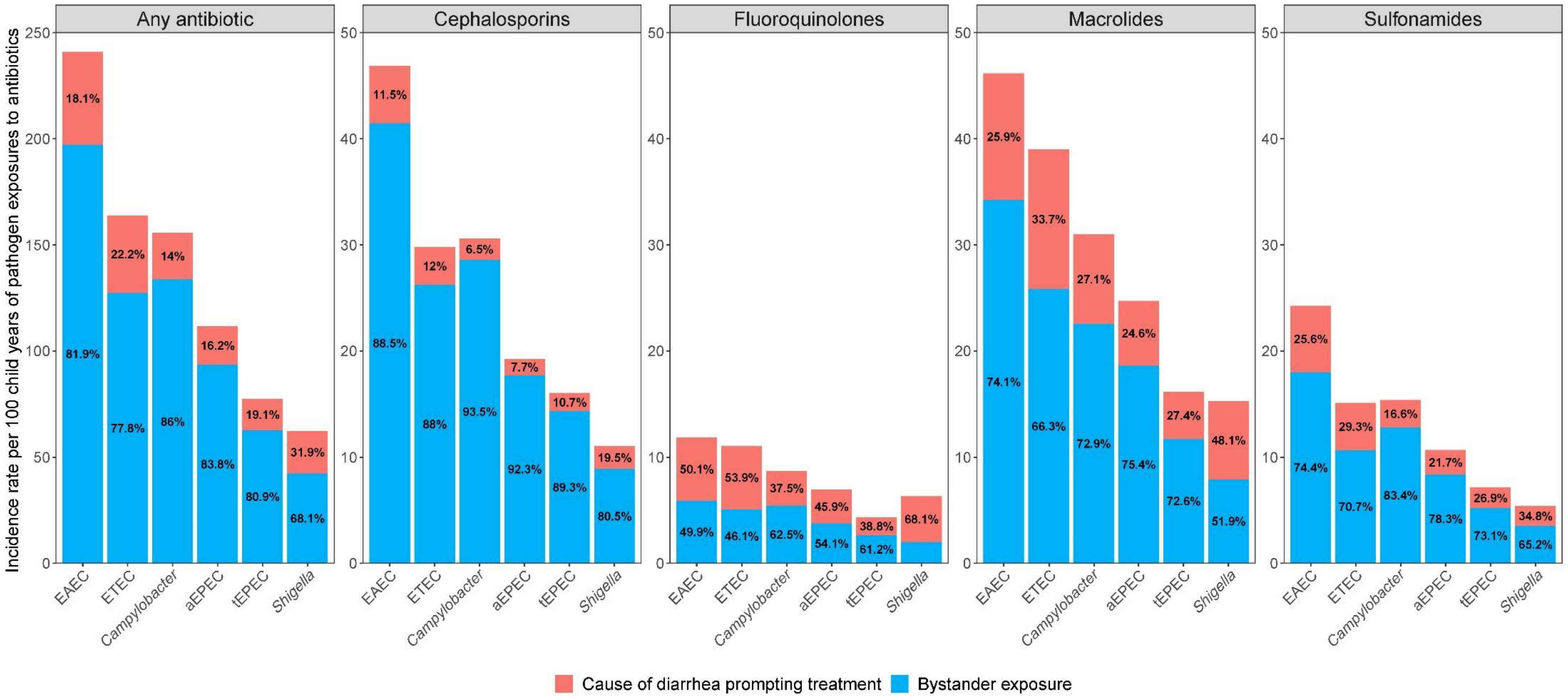

Table S3. Incidence rates of asymptomatically carried pathogen exposures to antibiotics by type of illness resulting in treatment among 1715 children in the MAL-ED cohort.

Type of illness	EAEC	*Campylobacter*	ETEC	aEPEC	tEPEC	*Shigella*	Any asymptomatically carried pathogen
Acute lower respiratory infection	29.4 (26.9, 32.1)	20.7 (18.2, 23.2)	15.7 (13.9, 17.5)	10.9 (9.6, 12.3)	9.2 (7.9, 10.6)	5.4 (4.5, 6.4)	91.3 (83.7, 99.1)
Upper respiratory infection	86.3 (82.2, 90.8)	52.2 (48.9, 55.8)	56.6 (52.8, 60.5)	40.6 (37.8, 43.7)	26.3 (24.1, 28.6)	18.1 (16.2, 20.1)	280.0 (266.6, 295.5)
Dysentery	6.2 (5.2, 7.3)	3.0 (2.4, 3.7)	3.5 (2.7, 4.3)	2.3 (1.8, 2.9)	1.3 (1.0, 1.8)	0.9 (0.5, 1.3)	17.3 (14.6, 20.1)
Diarrhea	51.7 (48.8, 55.0)	33.4 (30.8, 36.1)	33.3 (30.9, 35.9)	22.5 (20.5, 24.3)	16.7 (15.0, 18.4)	8.4 (7.1, 9.7)	166.0 (156.5, 175.7)
Other cause	55.9 (52.6, 59.5)	37.9 (34.7, 41.2)	37.8 (35.3, 40.6)	27.7 (25.5, 29.8)	16.8 (15.3, 18.5)	13.3 (12.0, 14.7)	189.5 (179.4, 200.1)

Data are incidence rates per 100 child years with 95% confidence intervals (CIs). These data also reported in Figure 3. EAEC = enteroaggregative *Escherichia coli.* ETEC= enterotoxigenic *Escherichia coli*. aEPEC = atypical enteropathogenic *Escherichia coli*. tEPEC = typical enteropathogenic *Escherichia coli*.

Figure S6. Incidence rates per 100 child years of enteric pathogen exposures to fluoroquinolones and macrolides and the proportion of exposures due to type of illness among 1715 children in the MAL-ED cohort.

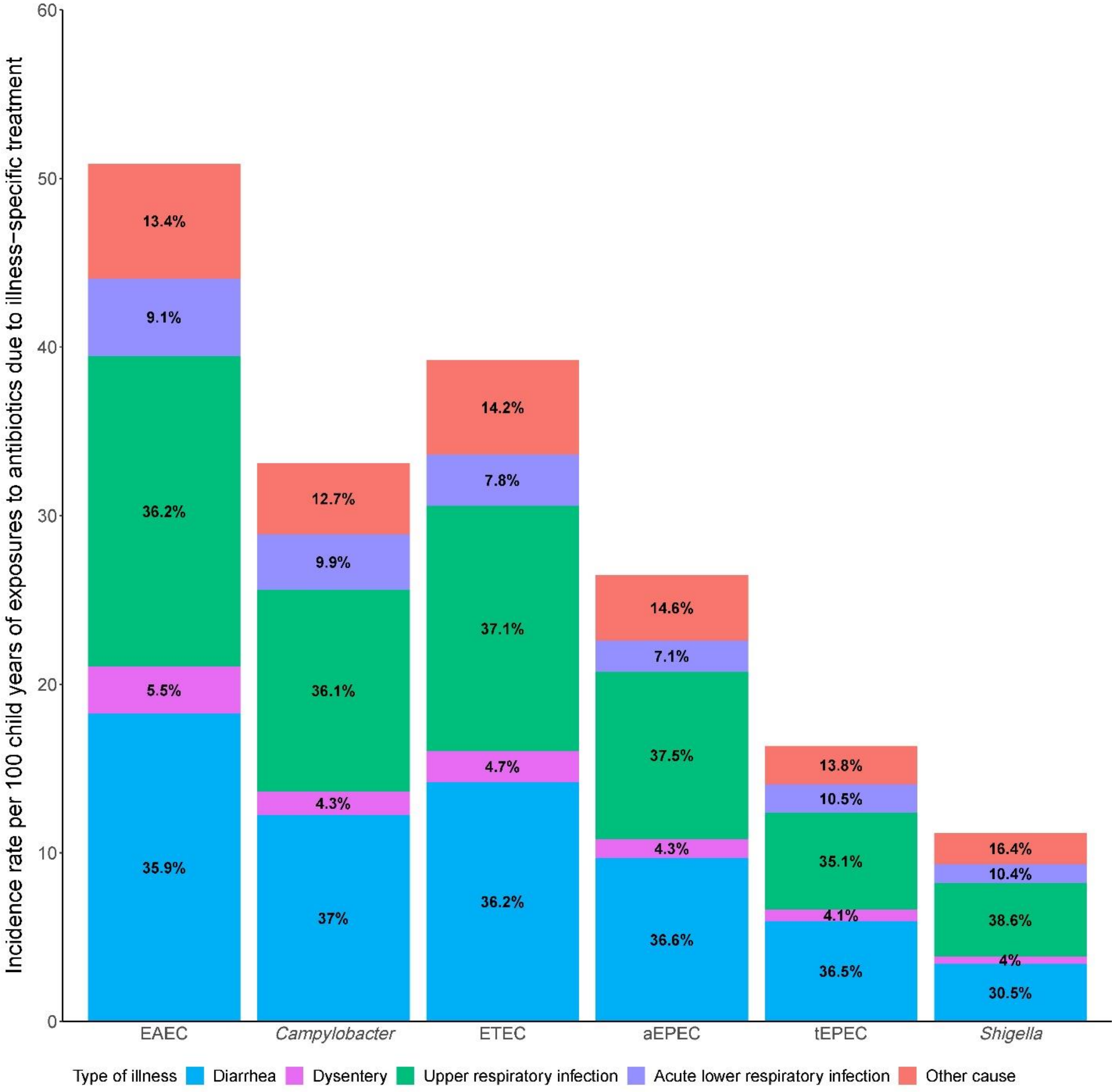

Table S4. Incidence rates of asymptomatically carried pathogen exposures to fluoroquinolones or macrolides by type of illness resulting in treatment among 1715 children in the MAL-ED cohort.

Type of illness	EAEC	*Campylobacter*	ETEC	aEPEC	tEPEC	*Shigella*	Any asymptomatically carried pathogen
Acute lower respiratory infection	4.6 (3.8, 5.5)	3.3 (2.5, 4.1)	3.1 (2.4, 3.8)	1.9 (1.4, 2.4)	1.7 (1.3, 2.2)	1.2 (0.8, 1.6)	15.7 (13.3, 18.5)
Upper respiratory infection	18.4 (16.8, 20.0)	12.0 (10.7, 13.4)	14.5 (13.0, 16.0)	9.9 (8.7, 11.1)	5.7 (4.9, 6.6)	4.3 (3.6, 5.1)	64.9 (59.8, 69.6)
Dysentery	2.8 (2.1, 3.5)	1.4 (1.0, 1.9)	1.8 (1.3, 2.4)	1.1 (0.8, 1.5)	0.7 (0.4, 0.9)	0.5 (0.2, 0.7)	8.3 (6.4, 10.1)
Diarrhea	18.3 (16.7, 19.9)	12.2 (10.7, 13.8)	14.2 (12.7, 15.7)	9.7 (8.6, 10.9)	6.0 (5.1, 6.9)	3.4 (2.7, 4.1)	63.8 (58.5, 68.8)
Other cause	6.8 (5.8, 7.7)	4.2 (3.5, 5.0)	5.6 (4.8, 6.4)	3.9 (3.2, 4.6)	2.3 (1.7, 2.8)	1.8 (1.4, 2.4)	24.5 (21.7, 27.3)

Data are incidence rates per 100 child years with 95% confidence intervals (CIs). These data also reported in Figure S5. EAEC = enteroaggregative *Escherichia coli*. ETEC= enterotoxigenic *Escherichia coli*. aEPEC = atypical enteropathogenic *Escherichia coli*. tEPEC = typical enteropathogenic *Escherichia coli*.

Table S5. Frequency and incidence rate ratios for the total number of times asymptomatically carried bacterial pathogens were exposed to any antibiotic among 1715 children in the MAL-ED cohort

Characteristic	Overall (N=1715[a])	Incidence Rate Ratio (95% CI) Individual Models[b]	Incidence Rate Ratio (95% CI) Multivariable Model[c]
Child characteristics			
Female (n, %)	841 (49.0)	0.86 (0.84, 0.89)	0.87 (0.84, 0.89)
Enrollment WAZ (mean, SD)	-0.80 (1.09)	1.01 (0.99, 1.02)	1.01 (1.00, 1.02)
Enrollment LAZ[d] (mean, SD)	-0.88 (1.05)	1.00 (0.98, 1.01)	--
Days exclusively breastfed in month increments[e] (mean, SD)	2.62 (1.92)	0.98 (0.97, 0.99)	0.98 (0.97, 0.99)
Sociodemographics			
WAMI score in 0.5 increments (mean, SD)	1.14 (0.45)	0.90 (0.86, 0.94)	--
Household income (≥150 USD) (n, %)	707 (41.2)	0.98 (0.95, 1.01)	0.98 (0.95, 1.01)
Maternal education (≥6 years)[f] (n, %)	1398 (81.5)	1.03 (1.00, 1.07)	1.05 (1.01, 1.09)
Maternal age in 5 year increments[g] (mean, SD)	5.26 (1.18)	1.00 (0.99, 1.02)	1.00 (0.99, 1.01)
Water and sanitation			
Improved source of drinking water (n, %)	1544 (90.0)	0.95 (0.89, 1.01)	0.95 (0.89, 1.01)
Treated water (n, %)	301 (17.6)	0.94 (0.91, 0.98)	0.94 (0.90, 0.98)
Access to improved latrine (n, %)	1237 (72.1)	0.91 (0.88, 0.95)	0.92 (0.89, 0.96)
Site (n, %)			
Dhaka, Bangladesh	210 (12.2)	--	--
Fortaleza, Brazil	165 (9.6)	0.03 (0.02, 0.03)	0.02 (0.02, 0.03)
Vellore, India	227 (13.2)	0.41 (0.39, 0.43)	0.37 (0.35, 0.39)
Bhaktapur, Nepal	227 (13.2)	0.21 (0.20, 0.22)	0.20 (0.19, 0.21)
Loreto, Peru	194 (11.3)	0.61 (0.58, 0.63)	0.53 (0.50, 0.56)
Naushero Feroze, Pakistan	246 (14.3)	0.73 (0.71, 0.76)	0.65 (0.61, 0.69)
Venda, South Africa	237 (13.8)	0.05 (0.04, 0.05)	0.04 (0.04, 0.05)
Haydom, Tanzania	209 (12.2)	0.45 (0.43, 0.47)	0.37 (0.34, 0.40)

LAZ = length for age z score. SD = standard deviation. USD = United States dollar. WAMI = Water, Assets, Maternal Education, Income. WAZ = weight for age z score. [a] Of whom 1595 were exposed to at least one course of antibiotics.
[b] Adjusted for site. [c] LAZ and WAMI removed from the model. The components of WAMI are household income, maternal education, improved water, and improved latrine. Therefore, WAMI score was removed from the multivariable model to avoid collinearity. [d] Pakistan excluded due to invalid measurements (N=246). [e] Days of exclusive breastfeeding includes all days. [f] Missing data (N=2) was imputed based on country average and then rounded to the nearest whole number. [g] Missing data (N=2) was based on site mean.

Table S6. Effect of class-specific antibiotic exposure in the last 30 days on the prevalence of resistance to the same drug class in 2434 cultured *E. coli* isolates.

Antibiotic class [a]	N (%) isolates exposed to antibiotics in last 30 days	N (%) isolates with resistance	Prevalence Ratio[b] (95% CI)
Macrolides	209 (8.6)	694 (28.5)	1.29 (1.13, 1.47)
Fluoroquinolones	41 (1.7)	552 (22.7)	1.18 (0.83, 1.70)
Sulfonamides	156 (6.4)	1617 (66.4)	1.02 (0.90, 1.15)
Cephalosporins	298 (12.2)	706 (29.0)	1.06 (0.93, 1.21)

[a] Antibiotic resistance for macrolides defined by azithromycin susceptibility testing, for fluoroquinolones by ciprofloxacin, for sulfonamides by trimethoprim/sulfamethoxazole, and for cephalosporins by ceftriaxone. [b] Adjusted for site, age, sex, WAMI index, and hospitalization in the last 90 days

Table S7. Classification of resistant *E. coli* for each antibiotic.

**Antibiotic**	**Cutoff for presence of resistant *E. coli*** [a]
Ceftriaxone	<23
Ciprofloxacin	<21
Trimethoprim/Sulfamethoxazole	<16
Azithromycin (E-test)	≥32

[a] Antibiotic susceptibility testing was performed using E-test for azithromycin and disk diffusion for all other antibiotics.
